# Modulation of *TP53* Expression by *Cosmos caudatus* (*Ulam raja*) in OSCC Cell Lines

**DOI:** 10.3390/ijms27146361

**Published:** 2026-07-17

**Authors:** Kelvin Sin-Hou Liaw, Sook-Luan Ng, Alida Mahyuddin, Norliwati Ibrahim, Eng-Wee Chua, Yee Xing You, Nurul Inaas Mahamad Apandi

**Affiliations:** 1 Department of Family Oral Health, Faculty of Dentistry, Universiti Kebangsaan Malaysia, Kuala Lumpur 50300, Malaysia; p138187@siswa.ukm.edu.my (K.S.-H.L.); alida@ukm.edu.my (A.M.); 2 Department of Craniofacial Diagnostics & Biosciences, Faculty of Dentistry, Universiti Kebangsaan Malaysia, Kuala Lumpur 50300, Malaysia; ngsookluan@ukm.edu.my (S.-L.N.); norliibrahim@ukm.edu.my (N.I.); 3 Faculty of Pharmacy, Universiti Kebangsaan Malaysia, Kuala Lumpur 50300, Malaysia; cew85911@ukm.edu.my; 4 Center for Healthy Aging & Wellness, Faculty of Health Sciences, Universiti Kebangsaan Malaysia, Kuala Lumpur 50300, Malaysia; youyeexing@ukm.edu.my

**Keywords:** oral squamous cell carcinoma (OSCC), *Cosmos caudatus*, *TP53*/p53, CD44, cytotoxicity

## Abstract

Oral squamous cell carcinoma (OSCC) is the most common malignancy of the head and neck, with current treatments offering limited improvement in survival outcomes. This has driven interest in natural compounds as potential therapeutic alternatives. *Cosmos caudatus* (CC), commonly known as “ulam raja,” possesses antioxidant and anticancer properties, but its effects on OSCC remain insufficiently explored. This study aimed to evaluate the cytotoxic effects of CC extract and its impact on *TP53*/p53 and *CD44* expression in an OSCC cell line in vitro. Cells were characterised using immunocytochemistry (ICC) and treated with CC extract (10–80 mg/mL). Cell viability was assessed using the MTT assay, while *TP53* and *CD44* gene expression were analysed via RT-qPCR. p53 protein levels were quantified using ELISA, and ICC was performed to assess protein expression. Results: CC demonstrated significant cytotoxicity with an IC_50_ of 58 mg/mL (*p* < 0.05). Treatment resulted in significant downregulation of *TP53*/p53 at both gene and protein levels (*p* < 0.05), alongside increased CD44 mRNA expression but reduced protein staining. CC may therefore have potential as a plant-derived adjunctive therapeutic agent, although further studies are required to elucidate its mechanisms and clinical applicability.

## 1. Introduction

Oral squamous cell carcinoma (OSCC) is a malignant neoplasm arising from the mucosal epithelium and characterised by squamous differentiation, as defined by the World Health Organisation (WHO). It represents 90% of oral cavity cancers and remains a significant global health burden, ranking among the sixteenth most common malignancies worldwide [1,2]. Despite advances in diagnostic and therapeutic approaches, OSCC continues to exhibit poor clinical outcomes, with a five-year survival rate of approximately 50–60% [3]. This is largely attributed to its aggressive biological behaviour, including local invasion, high recurrence rates, and metastatic potential.

The epidemiology of OSCC demonstrates marked geographical and demographic variation. Higher incidence rates are reported in South and Southeast Asia, particularly in populations with prevalent exposure to established risk factors such as tobacco smoking, alcohol consumption, and betel nut chewing [4,5]. These carcinogens act synergistically to induce chronic inflammation, oxidative stress, and cumulative deoxyribonucleic acid (DNA) damage, ultimately disrupting cellular regulatory mechanisms [6,7]. In addition to these traditional risk factors, emerging evidence highlights the role of viral infections, particularly human papillomavirus (HPV), as well as other contributing factors such as poor oral hygiene and chronic mucosal irritation [8,9]. Notably, a rising incidence of OSCC has been observed among younger individuals and non-smokers, suggesting the involvement of alternative molecular and environmental determinants [10,11,12,13,14].

At the molecular level, OSCC pathogenesis is a multifactorial process driven by genetic and epigenetic alterations that disrupt key signalling pathways regulating cell proliferation, apoptosis, and differentiation. Among these, the tumour suppressor gene *TP53* plays a central role in maintaining genomic stability by regulating DNA repair, cell cycle arrest, and apoptosis [6]. *TP53* is one of the most frequently mutated genes in OSCC, and its dysregulation is associated with impaired apoptotic responses, tumour progression, and resistance to chemotherapy and radiotherapy [15,16,17]. The protein product, p53, is widely recognised as the “guardian of the genome” and its altered expression has been correlated with poor prognosis and reduced therapeutic response in OSCC patients [18].

In parallel, increasing attention has been directed towards cancer stem cells (CSCs), a subpopulation of tumour cells with self-renewal and differentiation capabilities that contribute to tumour heterogeneity, therapeutic resistance, and disease recurrence [19,20]. CD44, a transmembrane glycoprotein involved in cell adhesion, migration, and signalling, is one of the most extensively studied CSC markers in OSCC [21,22]. Overexpression of *CD44* has been associated with enhanced tumour aggressiveness, increased metastatic potential, and resistance to both chemotherapy and radiotherapy [23,24,25]. However, its role as a prognostic marker remains variable, likely due to tumour heterogeneity and differences in isoform expression [23,24]. Nonetheless, targeting both apoptotic pathways and CSC-associated mechanisms is increasingly recognised as a promising strategy for improving OSCC treatment outcomes.

Current management of OSCC involves a multimodal approach, including surgery, radiotherapy, chemotherapy, targeted therapy, and immunotherapy [26]. While surgical intervention remains the cornerstone of treatment, it is often associated with significant functional and aesthetic morbidity, adversely affecting patients’ quality of life [27]. Adjuvant therapies, including platinum-based chemotherapy and radiotherapy, are frequently accompanied by systemic and local toxicities, as well as the development of treatment resistance [28,29]. Although newer approaches such as targeted therapy and immune checkpoint inhibitors have shown potential, their clinical benefits remain limited by cost, accessibility, and variable response rates [30,31]. These limitations underscore the need for alternative or adjunctive therapeutic strategies that are both effective and less toxic.

In recent years, natural plant-derived compounds have gained increasing attention as potential anticancer agents due to their ability to modulate multiple cellular pathways while exhibiting lower toxicity profiles [32]. Among these, *Cosmos caudatus* (CC), commonly known as “*Ulam raja*,” is a medicinal plant widely consumed in Southeast Asia [33]. CC is rich in bioactive compounds, particularly flavonoids such as quercetin, catechin, rutin, and proanthocyanidins, which possess strong antioxidant, anti-inflammatory, and anticancer properties [34,35,36,37,38]. These compounds have been shown to regulate oxidative stress, modulate signalling pathways, and induce apoptosis in cancer cells.

Epidemiological studies have demonstrated that dietary patterns rich in fruits, vegetables, and plant-derived antioxidants are associated with a lower risk of cancers [39]. Conversely, diets high in processed foods, saturated fats, and alcohol consumption have been linked to increased cancer incidence [40,41]. Although direct epidemiological evidence linking CC consumption to reduced OSCC incidence remains limited, its widespread dietary use and established biological activities support investigation into its potential anticancer properties.

Previous studies have demonstrated that CC exhibits cytotoxic and pro-apoptotic effects in various cancer models, including breast, colon, cervical, and leukaemia cell lines [42,43]. The anticancer activity of CC is primarily attributed to its ability to induce apoptosis via the intrinsic mitochondrial pathway, involving modulation of pro-apoptotic and anti-apoptotic proteins, activation of caspase cascades, and DNA fragmentation [44,45,46]. These processes are often mediated through p53-dependent signalling pathways, highlighting the potential role of *TP53* in the mechanism of action of CC. In addition, CC-derived flavonoids have been reported to induce cell cycle arrest at key checkpoints and inhibit tumour progression through modulation of signalling pathways such as PI3K/AKT/mTOR [47,48,49].

Despite these promising findings, current evidence remains limited in several aspects. Most studies investigating the anticancer effects of CC are confined to non-oral cancer cell lines and in vitro models, with considerable variability in extraction methods and experimental conditions [42,50]. More importantly, there is a lack of comprehensive investigation into its effects on OSCC, particularly with respect to key molecular pathways involving both tumour suppressor genes and cancer stem cell markers. While apoptosis-related mechanisms have been relatively well explored, the influence of CC on CSC-associated pathways, including *CD44* expression, remains largely unclear.

Given the critical roles of *TP53*/p53 in apoptosis and *CD44* in cancer stem cell maintenance and therapeutic resistance, investigating the dual modulation of these pathways represents an important area of research. Understanding how CC influences both apoptotic and stemness-related mechanisms may provide valuable insights into its potential as a plant-derived anticancer agent.

Therefore, this study aims to evaluate the cytotoxic effects of CC extract on OSCC cells and to elucidate its molecular impact on *TP53*/p53 and *CD44* expression at both gene and protein levels. By integrating cellular and molecular analyses, this study seeks to address an important gap in the current literature and to explore the potential of CC as a novel therapeutic or adjunctive strategy in OSCC management.

## 2. Results

### 2.1. Nutrient Composition of CC Extract

The nutrient composition of CC extract was analysed to determine its nutritional profile and potential bioactive components (Table 1).

The CC extract demonstrated high carbohydrate and ash content, indicating the presence of energy sources and essential minerals such as iron, potassium, sodium, and zinc. Phytochemical analysis identified flavonoids, quercetin, and polyphenols, along with antioxidant vitamins (A, C, and E). These components collectively suggest strong antioxidant capacity and potential anticancer activity.

### 2.2. OSCC Cell Characterisation

**Figure 1:**
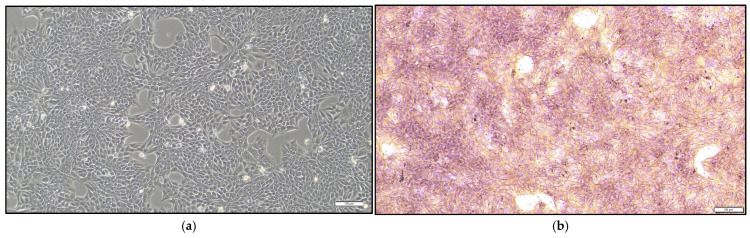
OSCC cells viewed under a phase-contrast inverted microscope and light microscope at 100× magnification. (**a**) Unstained OSCC cells show typical malignant morphology. (**b**) OSCC cells stained with CD44, demonstrating predominantly membranous brown staining. Scale bar = 100 µm.

Unstained OSCC cells exhibited characteristic malignant morphology, including polygonal to spindle-shaped cells, irregular borders, and loss of contact inhibition (Figure 1a).

Immunocytochemistry (ICC) analysis revealed strong membranous CD44 expression, with prominent brown staining in the majority of cells. Cytological features such as nuclear pleomorphism, hyperchromatism, and increased nuclear-to-cytoplasmic ratio further confirmed the malignant phenotype (Figure 1b).

### 2.3. Cytotoxicity Assay (MTT Assay)

#### 2.3.1. Effects of Cisplatin

**Figure 2:**
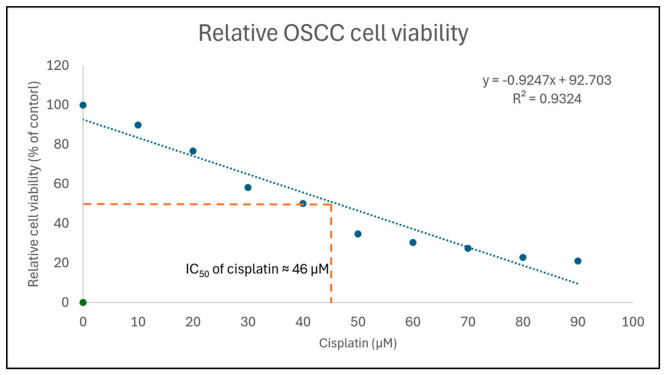
OSCC relative cell viability at different concentrations of cisplatin.

Cisplatin induced a dose-dependent decrease in cell viability, reducing viability to 21% at 90 µM. Statistical analysis showed significant differences compared with the negative control (*p* < 0.05). The IC_50_ was approximately 46 µM, derived from the dose–response curve shown in Figure 2.

#### 2.3.2. Effects of CC Extract

**Figure 3:**
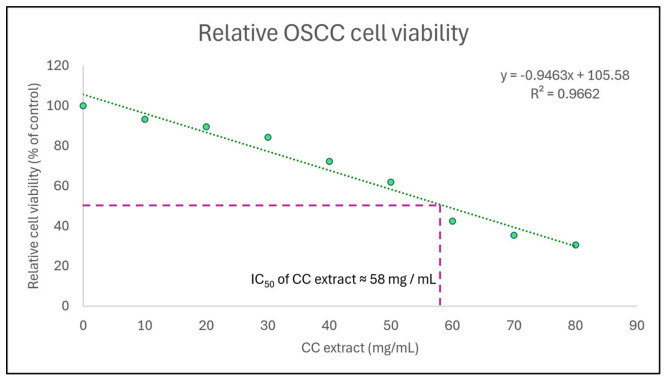
OSCC relative cell viability of different concentrations of CC extract.

Similarly, CC extract demonstrated dose-dependent cytotoxicity, with cell viability reduced to 31% at 90 mg/mL. Significant differences were observed across treatment groups (*p* < 0.05). The IC_50_ was estimated at 58 mg/mL, based on the dose–response curve equation shown in Figure 3.

### 2.4. Genomic Assay (RT-qPCR)

**Figure 4:**
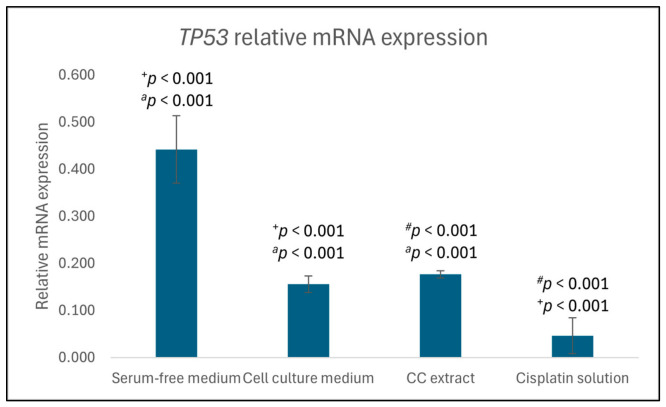
Relative mRNA expression of *TP53* gene across different treatment groups. Data are presented as mean ± SEM (n = 6), ^#^ *p* < 0.001 vs. negative control (serum-free medium); ^+^ *p* < 0.001 vs. CC extract; ^a^ *p* < 0.001 vs. cisplatin solution.

RT-qPCR analysis showed that both CC extract (58 mg/mL) and cisplatin (46 µM) significantly downregulated *TP53* expression compared to the control (*p* < 0.001) (Figure 4).

**Figure 5:**
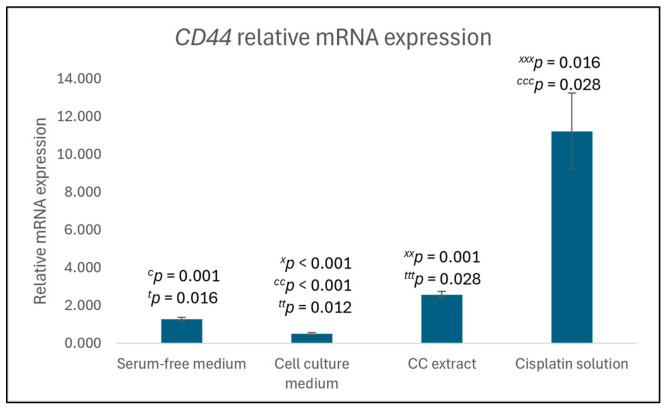
Relative mRNA expression of *CD44* gene between groups. Data are presented as mean ± SEM (n = 6), ^x^ *p* < 0.001, ^xx^ *p* = 0.001, ^xxx^ *p* = 0.016 vs. negative control (serum-free medium); ^c^ *p* = 0.001, ^cc^ *p* < 0.001, ^ccc^ *p* = 0.028 vs. CC extract; ^t^ *p* = 0.016, ^tt^ *p* = 0.012, ^ttt^ *p* = 0.028 vs. cisplatin solution.

**Figure 6:**
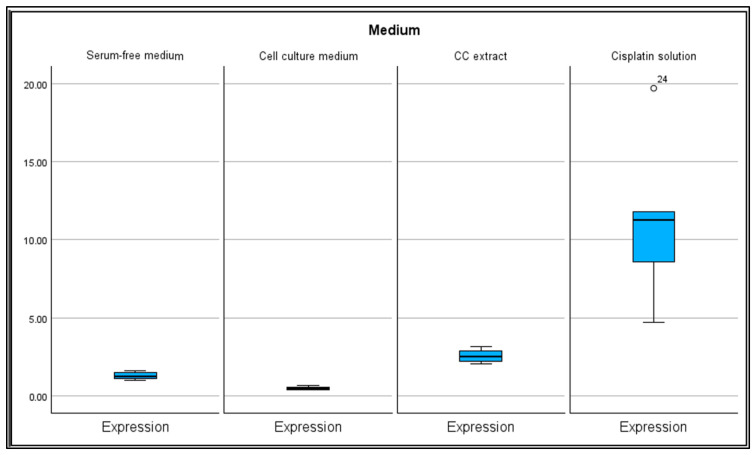
Box plot showing the distribution of *CD44* relative gene expression among the serum-free medium, cell culture medium, CC extract, and cisplatin solution groups. The central line represents the median, the box denotes the interquartile range, whiskers indicate the range excluding outliers, and circles represent outliers.

In contract, CC extract and cisplatin significantly upregulated CD44 gene expression compared to the control group (^xx^ *p* = 0.001; ^xxx^ *p* = 0.016) (Figure 5 and Figure 6).

### 2.5. Protein Assay (ELISA)

**Figure 7:**
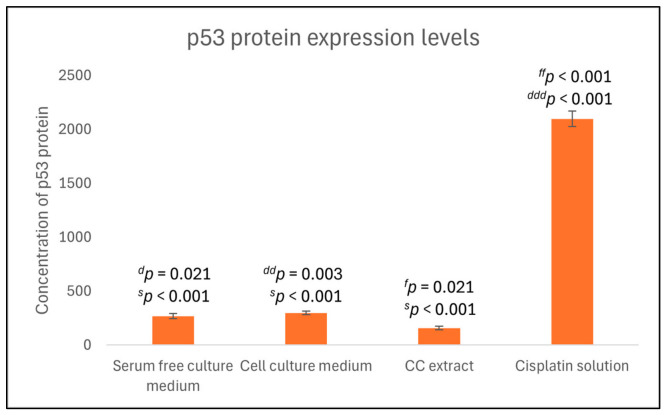
p53 protein expression levels between groups. Data are presented as mean ± SEM (n = 6), ^f^ *p* = 0.021, ^ff^ *p* < 0.001 vs. negative control (serum-free medium); ^d^ *p* = 0.021, ^dd^ *p* = 0.003, ^ddd^ *p* < 0.001 vs. CC extract; ^s^ *p* < 0.001 vs. cisplatin solution.

ELISA results demonstrated that CC treatment significantly reduced p53 protein levels compared to the control (*p* < 0.05). Conversely, cisplatin treatment significantly increased p53 protein expression (*p* < 0.05), consistent with its mechanism of inducing DNA damage (Figure 7).

### 2.6. Immunocytochemistry (ICC)

ICC was performed to evaluate the expression and localisation of p53 and CD44 in OSCC cells following treatment with CC extract and cisplatin. Staining intensity, localisation, and the proportion of positively stained cells were assessed under a light microscope across multiple representative fields. Three groups were analysed: negative control (serum-free medium), cisplatin-treated (46 µM), and CC-treated (58 mg/mL).

**Figure 8:**
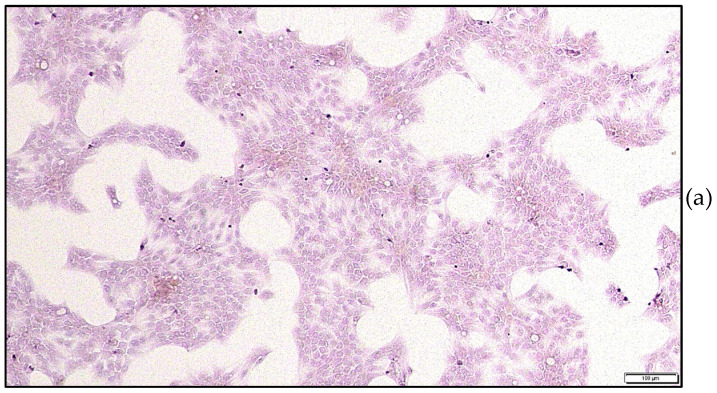

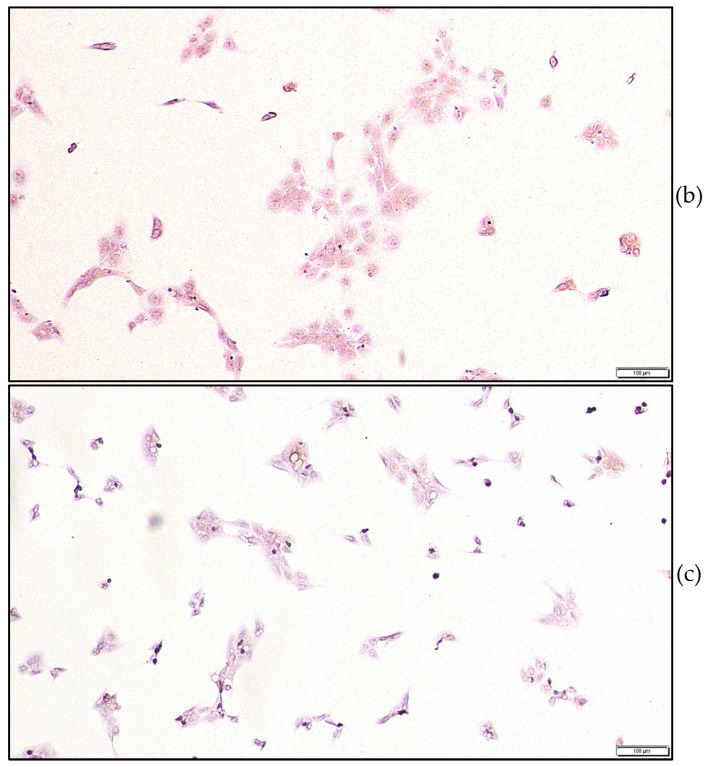
Immunocytochemical staining of p53 in OSCC cells observed under a light microscope at 100× magnification. (**a**) OSCC cells cultured in serum-free culture medium (negative control). (**b**) OSCC cells treated with cisplatin solution (positive control). (**c**) OSCC cells treated with CC extract. Scale bar = 100 µm.

**Figure 9:**
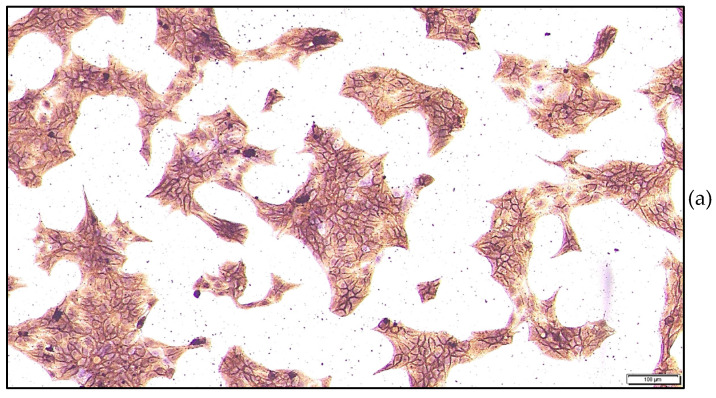

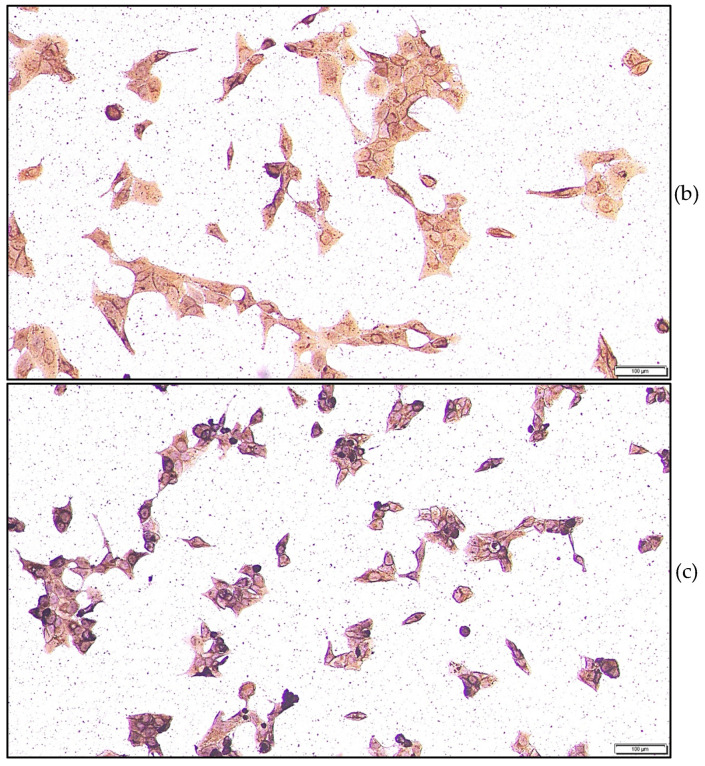
Immunocytochemical staining of CD44 in OSCC cells observed under a light microscope at 100× magnification. (**a**) OSCC cells cultured in serum-free culture medium (negative control). (**b**) OSCC cells treated with cisplatin solution (positive control). (**c**) OSCC cells treated with CC extract. Scale bar = 100 µm.

In the negative control group, OSCC cells demonstrated baseline nuclear p53 staining and strong, continuous membranous CD44 expression (Figure 8a and Figure 9a). Cells exhibited typical malignant morphology, including irregular shapes, nuclear pleomorphism, and dense, disorganised growth.

Cisplatin-treated cells showed markedly increased nuclear p53 staining, indicating enhanced p53 stabilisation following DNA damage (Figure 8b). Morphological features of cytotoxicity, including cell shrinkage, nuclear condensation, and reduced cell density, were evident. CD44 expression remained strongly membranous despite decreased cell numbers (Figure 9b).

In contrast, CC-treated cells exhibited reduced nuclear p53 staining compared to both control and cisplatin groups (Figure 8c), consistent with decreased *TP53* expression observed in RT-qPCR. CD44 membranous staining was also diminished (Figure 9c). Morphological changes, including cytoplasmic shrinkage and reduced confluence, were observed, indicating decreased cell viability and potential modulation of cancer stem cell-associated features.

## 3. Discussion

This study investigated the effects of CC extract on OSCC cells, focusing on cytotoxicity and modulation of *TP53*/p53 and *CD44* expression. The findings demonstrated that CC treatment reduced cell viability and significantly downregulated *TP53*/p53 expression at both gene and protein levels. These results suggest that CC may influence key regulatory pathways involved in apoptosis and cell cycle control in OSCC.

The following discussion contextualises these findings within existing literature, with emphasis on the potential molecular mechanisms underlying CC-mediated effects and their relevance to OSCC therapeutic development.

### 3.1. Nutritional and Phytochemical Composition of CC Extract

The biological effects of CC extract can be attributed to its complex composition of macronutrients, micronutrients, and bioactive phytochemicals. Although carbohydrates and mineral components do not directly exert strong anticancer effects, they contribute to the stability and bioavailability of active compounds. Carbohydrates may form molecular complexes with phenolic compounds, protecting them from degradation, while mineral elements support redox homeostasis through their role as enzymatic cofactors [51].

Antioxidant vitamins, including vitamins A, C, and E, further enhance the biological activity of CC. These compounds reduce oxidative stress by scavenging reactive oxygen species (ROS) and preventing oxidative DNA damage [52]. Vitamin C, in particular, has been shown to induce apoptosis and cell cycle arrest in OSCC models, partly through p53-associated pathways [53,54,55]. Similarly, α-tocopherol contributes to membrane stabilisation and modulation of cell proliferation [56].

Polyphenols, especially flavonoids such as quercetin, represent the most critical contributors to the anticancer potential of CC. These compounds exert antioxidant effects by neutralising ROS, chelating metal ions, and inhibiting oxidative enzymes [57,58]. Quercetin additionally enhances intracellular antioxidant defence systems, including glutathione and superoxide dismutase activity, thereby reducing oxidative stress [59,60,61]. Collectively, these components create a biochemical environment that supports anticancer activity, primarily through modulation of oxidative stress and apoptosis-related pathways.

### 3.2. Biological Responses of OSCC Cells to CC Extract and Cisplatin Treatments

#### 3.2.1. Effects on Cell Viability

CC extract demonstrated a dose-dependent reduction in OSCC cell viability, indicating clear antiproliferative activity. The gradual decline in metabolic activity suggests that CC exerts predominantly cytostatic effects, limiting proliferation rather than inducing rapid cytotoxicity. This observation is consistent with previous studies attributing the anticancer effects of CC to its phenolic and flavonoid constituents [62].

Although the IC_50_ of CC (58 mg/mL) was substantially higher than that of cisplatin (46 µM, equivalent 0.0138 mg/mL), this difference reflects the lower potency of crude plant extracts compared to purified chemotherapeutic agents [63]. Variability in IC_50_ values across OSCC cell lines, as reported in the literature, further highlights the influence of biological heterogeneity and methodological factors [64,65,66]. These include differences in cell density, passage number, assay conditions, and intrinsic genetic variability, underscoring the importance of experimental standardisation [67].

#### 3.2.2. Modulation of Gene Expression

CC treatment resulted in significant downregulation of *TP53* gene expression. Given that *TP53* is frequently mutated in OSCC, this reduction may reflect suppression of mutant p53 rather than inhibition of tumour-suppressive function. Mutant p53 proteins often accumulate abnormally and promote oncogenic processes, including proliferation and therapeutic resistance [68]. Therefore, decreased *TP53* expression may indicate disruption of mutant p53-driven pathways [69].

The antioxidant properties of CC may further contribute to this effect by reducing oxidative stress, thereby attenuating stress-induced p53 activation [70]. In contrast, cisplatin typically induces p53 via DNA damage; however, its limited effect in this study suggests the presence of dysfunctional or mutant p53 in the OSCC model [68].

The influence of serum supplementation introduces an important variable in interpreting *TP53* expression. In this study, OSCC cells cultured with FBS showed lower p53 expression compared with serum-free conditions. FBS contains various growth factors and bioactive components that support cell survival and maintain cellular homeostasis. Removal of serum exposes cells to nutrient deprivation and increased oxidative stress, resulting in elevated intracellular reactive oxygen species (ROS) levels [71,72,73]. As p53 is a key stress-responsive protein activated by oxidative damage and cellular stress signals, serum deprivation may stimulate *TP53* transcription as part of a protective cellular response [72,74]. Therefore, the increased *TP53* expression observed in the serum-free control group is likely a consequence of stress-induced activation of p53 signalling rather than a treatment-related effect.

In parallel, *CD44* expression was significantly upregulated following both CC and cisplatin treatment. *CD44* is a transmembrane glycoprotein involved in cell–cell and cell–extracellular matrix interactions and is a recognised CSC marker in OSCC, contributing to tumour initiation, progression, invasion, and therapeutic resistance [75]. The increase in *CD44* expression following CC treatment may reflect a compensatory stress response or selective survival of *CD44*-positive subpopulations with enhanced resistance [76]. Alternatively, CC may preferentially eliminate rapidly proliferating cells while sparing CSC-like populations, leading to relative enrichment of *CD44* expressing cells [77,78]. Similarly, cisplatin-induced stress has been shown to enrich or activate *CD44*-positive CSC-like cells, contributing to chemoresistance [79]. *CD44* overexpression is associated with enhanced DNA repair, survival signalling, and reduced drug sensitivity, and has been implicated in cisplatin resistance in OSCC [80]. Mechanistically, *CD44* promotes tumour aggressiveness through hyaluronan-mediated adhesion, increased extracellular vesicle release, and activation of survival pathways such as PI3K/Akt and MAPK [66].

However, despite the increase in *CD44* mRNA expression, ICC demonstrated reduced CD44 protein staining following CC treatment. This discrepancy suggests the involvement of post-transcriptional regulation and indicates that increased gene expression did not necessarily translate into increased protein expression.

#### 3.2.3. Alterations in Protein Expression

Consistent with gene expression findings, CC treatment significantly reduced p53 protein levels, indicating modulation of p53 related pathways. However, the present study did not directly assess apoptosis; therefore, the involvement of apoptotic mechanisms cannot be confirmed from the current data.

In contrast, cisplatin-treated cells exhibited a clear increase in p53 protein concentration, despite the observed reduction in TP53 mRNA expression. Cisplatin generates DNA crosslinks that stabilise p53 by preventing mouse double minute 2 homologueue (MDM2)-mediated ubiquitination and proteasomal degradation. Consequently, existing p53 protein accumulates within the cell even when TP53 transcription is reduced, leading to increased protein levels and activation of downstream pathways associated with cell cycle arrest and apoptosis [68].

However, in OSCC, where TP53 mutations are prevalent, the accumulation of p53 may not necessarily indicate restoration of tumour-suppressive function. Mutant p53 proteins can accumulate to high levels and contribute to gain-of-function oncogenic activities, including enhanced proliferation, invasion, and chemoresistance [81,82].

### 3.3. Immunocytochemical Profiling of OSCC Cells

Immunocytochemical analysis provided visual confirmation of molecular findings. CC-treated cells exhibited reduced nuclear p53 staining and decreased CD44 expression, alongside morphological changes indicative of apoptosis, including cell shrinkage and reduced confluence [83].

The reduction in CD44 protein expression suggests that CC may influence cancer stem cell characteristics, despite the observed increase in CD44 gene expression. This discrepancy may reflect post-transcriptional regulation or selective effects on specific cell subpopulations [84,85]. Overall, ICC findings reinforce the potential of CC to modulate both survival and stemness-related pathways in OSCC.

### 3.4. Implications for OSCC Therapeutic Development

The findings of this study demonstrate that CC exerts inhibitory effects on OSCC cells, as evidenced by reduced cell viability and downregulation of *TP53*/p53 at both gene and protein levels. These results support its potential anticancer activity, consistent with the recognised role of plant-derived phytochemicals as adjuncts in cancer therapy [86,87,88].

The reduction in p53 expression is particularly relevant, as OSCC commonly harbours mutant *TP53* with oncogenic gain-of-function properties that promote tumour progression and resistance [89]. Thus, the observed downregulation may reflect suppression of mutant p53 stabilisation rather than loss of tumour-suppressive activity, suggesting that CC interferes with aberrant p53-driven signalling [90,91].

These effects are likely mediated by CC’s bioactive phytochemicals, which are known to induce apoptosis, promote G0/G1 cell cycle arrest, and modulate oxidative stress. Their antioxidant activity may reduce ROS-mediated survival signalling, contributing to decreased *TP53* expression and reduced cell viability [92,93].

Overall, CC shows promise as a complementary agent in OSCC management. By modulating key molecular pathways, including mutant p53-associated mechanisms, it may enhance therapeutic efficacy and help address chemoresistance.

### 3.5. Limitations and Future Directions

Several limitations should be acknowledged. The MTT assay measures metabolic activity rather than direct cell viability, and complementary assays such as apoptosis (Annexin V/PI staining, caspase activation, TUNEL or Bax/Bcl-2 assays) and cell cycle analysis are recommended to provide a more complete evaluation of cell response [94].

The use of crude extract also limits identification of specific bioactive compounds responsible for the observed effects, highlighting the need for phytochemical isolation and fractionation studies.

Although changes in *TP53*/p53 and *CD44* expressions were observed, RT-qPCR and ELISA do not distinguish between wild-type and mutant p53. Given the prevalence of *TP53* mutations in OSCC, future studies should incorporate mutation-specific analysis and functional assays to improve mechanistic clarity [95,96].

The study was also limited to a single OSCC cell line. Expanding research into multiple OSCC cell lines and including normal oral epithelial cells would help confirm selectivity and improve translational relevance. More detailed mechanistic work, including apoptosis profiling, cell cycle analysis, and migration or invasion assays would help clarify the functional impact of CC on tumour behaviour. Investigation into downstream signalling pathways and oxidative stress markers may further elucidate its molecular mechanisms [36,64,65,66].

Finally, in vivo validation is necessary to confirm efficacy, safety, and pharmacokinetic behaviour, and to support the potential development of CC as an adjunctive therapeutic agent in OSCC management.

## 4. Materials and Methods

### 4.1. Preparation of CC Extract

Aqueous CC extract was obtained commercially from Bionutricia Manufacturing Sdn. Bhd., Malaysia. The extract was prepared via ultrasonic-assisted extraction at 50 °C, followed by filtration, evaporation, and spray drying. The powder was packed in an aluminium foil plastic zipper bag and kept in a 4 °C refrigerator until further use.

Macronutrients (carbohydrate, protein, fat), micronutrients (vitamins A, C, E), ash, moisture, total flavonoids, quercetin, and polyphenols content in CC were analysed. All analysis procedures were carried out by accredited food analysis company, ALS Global Sdn. Bhd. and Unipeq Sdn. Bhd., using in-house methods developed based on AOAC International, and Pearson’s The Chemical Analysis of Food guidelines.

A stock solution (500 mg/mL) was prepared in Dulbecco’s Modified Eagle Media F-12 (DMEM/F-12) supplemented with L-Glutamine, sodium pyruvate, and HEPES liquid (Nacalai Tesque, Kyoto, Japan) and sterilised using a 0.2 µm sterile polyethersulfone (PES) syringe filter (Greenmall, Taichung City, Taiwan). The prepared stock solution was stored at 4 °C until further use.

### 4.2. Cell Culture

The human OSCC cell line OECM-1 category SCC180 was purchased from Merck, Germany. The OSCC cell line was derived from the surgical resection of a primary tumour from gingival epidermal carcinoma [97]. The OSCC cell line was cultured in DMEM/F-12 supplemented with 10% foetal bovine serum (FBS; Capricorn, Ebsdorfergrund, Germany) and 1% penicillin-streptomycin. Cells were maintained at 37 °C in a humidified 5% CO_2_ incubator and used between passages 4–6. Subculturing was performed at approximately 90% confluency using trypsin/ethylene-diaminetetraacetic acid (EDTA) solution (Sigma, St. Louis, MO, USA).

### 4.3. Cell Characterisation

Cell characterisation was performed using ICC to evaluate CD44 protein expression in the OSCC cell line. Approximately 6 × 10^4^ cells were seeded into 4-well chamber slides (Biologix, Lenexa, KS, USA) and incubated for 24 h at 37 °C in a humidified atmosphere containing 5% CO_2_ to allow cell attachment. Cell morphology and attachment were confirmed using a phase-contrast inverted microscope (Olympus, Tokyo, Japan). Following cell attachment, the spent culture medium was replaced with 100 µL of fresh medium per well, and cells were further incubated until approximately 90% confluency was reached.

Once confluency was achieved, cells were fixed with 4% paraformaldehyde (Elabscience, Houston, TX, USA) for 24 h, followed by permeabilisation with cold acetone at −20 °C for 5 min. Antigen retrieval was performed using EnVision FLEX Target Retrieval Solution (high pH; Dako, USA) in a PT Link system at 97 °C for 20 min.

Endogenous peroxidase activity was blocked using EnVision FLEX Peroxidase-Blocking Reagent (Dako, Carpinteria, CA, USA) for 5 min at room temperature. After washing with EnVision FLEX Wash Buffer (Dako, Hamburg, Germany), cells were incubated with primary antibody, rabbit recombinant monoclonal anti-CD44 primary antibody (Abcam, Cambridge, UK), and secondary antibody, EnVision FLEX/HRP secondary antibody (Dako, Germany), containing goat anti-rabbit immunoglobulin for 30 min at room temperature. Washing steps (3 min each) were performed between incubations with EnVision FLEX Wash Buffer.

Immunoreactivity was visualised using EnVision FLEX DAB+ Chromogen (Dako, Germany). Cells were counterstained with haematoxylin, dehydrated, and mounted with coverslips. Stained cells were examined under a light microscope (Olympus, Tokyo, Japan) to assess CD44 expression.

### 4.4. Preparation of Controls Solution

Cisplatin was used as the positive control, while serum-free culture medium served as the negative control in all assays. Cisplatin (TargetMol, Wellesley Hills, MA, USA) was supplied in powder form and prepared as a stock solution by dissolving 3 mg in 1 mL of distilled water to obtain a final concentration of 3 mg/mL. The solution was sterilised using a 0.2 µm syringe filter (25 mm), aliquoted, and stored at −20 °C until further use [98].

Serum-free culture medium was prepared by supplementing DMEM/F-12 with 1% penicillin–streptomycin under sterile conditions and stored at 4 °C until further use.

Complete cell-culture medium consisting of DMEM/F-12 supplemented with 10% foetal bovine serum (FBS) and 1% penicillin–streptomycin was used as the standard culture condition for routine cell maintenance. In contrast, serum-free culture medium was used as the experimental negative control during treatment assays because both cisplatin and CC extract were prepared and diluted in serum-free medium. This ensured that all treatment groups were exposed to identical experimental conditions, differing only in the presence or absence of the test compounds.

### 4.5. Cell Preparation and Treatment

#### 4.5.1. Cell Counting and Seeding

Cell preparation was performed under sterile conditions. Cells harvested from T-25 flasks were trypsinised, resuspended in 9 mL of complete culture medium, and mixed with 0.5% trypan blue solution (Nacalai Tesque, Kyoto, Japan) at a ratio of 9:1 (*v*/*v*). Viable cells were counted using a haemocytometer (Neubauer, Hirschmann, Germany) under a phase-contrast inverted microscope.

Cell concentration was calculated using the standard formula [99]:

Total number of viable cells = Average cell counts per grid × Dilution factor × 10^4^ × Volume of suspension, (1)

A total of 5 × 10^3^ viable cells in 100 µL of culture medium were seeded into each well of a 96-well plate. Peripheral wells were filled with sterile phosphate-buffered saline (PBS) to minimise edge effects. The plate was incubated at 37 °C in a humidified atmosphere with 5% CO_2_ for 24 h to allow cell attachment before treatment.

#### 4.5.2. Treatment with Test and Control Solutions

Following incubation, cell attachment was confirmed microscopically. The spent culture medium was removed, and cells were treated with either cisplatin or CC extract.

The cisplatin stock solution (3 mg/mL) was serially diluted in serum-free culture medium to obtain concentrations ranging from 10 to 90 µM. The CC extract stock solution (500 mg/mL) was similarly diluted in serum-free culture medium to obtain concentrations ranging from 10 to 90 mg/mL. Treatments were applied in triplicate wells.

After treatment, plates were incubated at 37 °C in a humidified 5% CO_2_ atmosphere for 72 h, in accordance with ISO 10993-5 [100] guidelines for in vitro cytotoxicity testing.

### 4.6. Cytotoxicity Assay (MTT)

Thiazolyl blue tetrazolium bromide (MTT; Sigma, USA) stock solution (5 mg/mL) was prepared using distilled water, filtered, and stored at −20 °C protected from light [101]. After treatment, 10 µL MTT (0.5 mg/mL final concentration) was added to each well and incubated for 4 h. Formazan crystals were dissolved in 100 µL dimethylsulfoxide (DMSO; Alfa Aesar, Ward Hill, MA, USA), and absorbance was measured at 570 nm using a microplate spectrophotometer (Thermo Fisher, Waltham, MA, USA).

Cell viability was calculated relative to the negative control after blank correction, using the following formula, in accordance with ISO 10993-5 (2009) guidelines for in vitro cytotoxicity testing:

(2) $$\mathrm{Cell}\;\mathrm{viability}\;\left(\%\right)=\frac{({\mathrm{OD}}_{570}\;\mathrm{treatment}\;-\;{\mathrm{OD}}_{570}\;\mathrm{blank})}{({\mathrm{OD}}_{570}\;\mathrm{negative}\;\mathrm{control}\;-\;{\mathrm{OD}}_{570}\;\mathrm{blank})}\times \;100$$

The serum-free medium group served as the negative control and reference group for normalisation of percentage cell viability.

### 4.7. Gene Expression Analysis (RT-qPCR)

Total RNA was extracted using the innuPREP RNA Mini Kit 2.0 (IST Innuscreen, Berlin, Germany) according to manufacturer’s protocols. Cells (3 × 10^5^) were seeded in 6-well plates, and treated with cell culture medium, serum-free culture medium, CC solution, or cisplatin solution for 72 h. The concentration of CC solution and cisplatin solution were determined based on the results obtained from cytotoxicity assay. Cells were lysed with lysis buffer, and the lysate was applied to spin columns with 70% ethanol addition, followed by sequential washing and centrifugation steps to purify the RNA. The RNA was subsequently eluted using RNase-free water. The RNA concentration and purity were determined using a Nanodrop Spectrophotometer ND2000 (Thermo Scientific, USA) and used for downstream cDNA synthesis.

SensiFAST cDNA kit (Bioline, London, UK) was used to synthesise complementary DNA from 200 ng of total RNA, following the manufacturer’s protocols. The reverse transcription was initiated with 10 min at 25 °C for primer annealing, 15 min at 42 °C for reverse transcription, 5 min at 85 °C for reaction termination and finally cooling to 4 °C.

Glyceraldehyde-3-phosphate dehydrogenase (*GAPDH*) was used as the housekeeping gene for data normalisation and *TP53* and *CD44* gene were used as the target gene. The oligonucleotide primer used was designed using National Centre for Biotechnology Information (NCBI) Primer Blast using Primer Output 3 software and synthesised by Bio Basic, Canada. The oligonucleotide primer sequences were shown in Table 2.

qPCR was performed using SensiFAST SYBR No-Rox kit (Bioline, UK), following the manufacturer’s protocols, in Real-Time PCR Detection System (185-5096) (Bio-Rad, Hercules, CA, USA). The thermal cycling conditions were 95 °C (2 min), followed by 40 cycles of denaturation at 95 °C for 5 s, annealing at 60 °C for 10 s, and extension at 72 °C for 20 s. At the end of amplification cycles, a melt curve analysis was performed to verify the specificity of amplification. The threshold cycle (Ct) values obtained for *TP53* and *CD44* genes were normalised to the Ct values of *GAPDH* gene, and the relative expression levels were determined using the comparative threshold cycle equation as follows [102]:

(3) $$\mathrm{Relative}\;\mathrm{expression}\;\mathrm{level}\;=\;2^{-\Delta \Delta \mathrm{Ct}}$$

where ΔΔCt represents the normalised Ct difference between treated and control samples in the target group relative to *GAPDH* group. All experiments were performed in triplicate.

For relative quantification analysis, the serum-free medium group was used as the calibrator control for normalisation after internal normalisation to GAPDH expression.

### 4.8. Protein Analysis (ELISA)

p53 protein levels were quantified using a human total p53 ELISA kit (Finetest, Wuhan, China) following the manufacturer’s protocols. Supernatants collected after treatment were centrifuged and the clarified supernatants were used for analysis.

Standards (78.125–5000 pg/mL) were prepared via serial dilution. Samples and standards (100 µL) were added to the ELISA plate and incubated at 37 °C for 90 min, followed by sequential incubation with biotin-labelled antibody and HRP-Streptavidin Conjugate at 37 °C for 60 min and 30 min, respectively. After washing steps, tetramethylbenzidine (TMB) substrate was added and incubated in the dark at 37 °C for 15 min. Finally, the reaction was stopped with stop solution prior to measurement at 450 nm. Protein concentrations were determined from the standard curve. Protein expression levels obtained from treated groups were interpreted relative to the serum-free medium control group.

### 4.9. Immunocytochemistry (ICC)

ICC was performed to assess p53 and CD44 protein expressions. Serum-free medium group was used as the baseline reference for qualitative comparison of staining intensity and protein localisation patterns. Cell cultivation, fixation, rehydration, antigen retrieval, and CD44 immunocytochemistry were carried out following the previously described cell characterisation protocol.

Cells were first incubated with *ultra*View Universal DAB Inhibitor containing 3% hydrogen peroxide solution (Ventana, Tucson, AZ, USA) at room temperature for 5 min, then with primary antibody, monoclonal mouse anti-human p53 protein (Dako, Germany), followed by secondary antibody, *ultra*View Universal HRP Multitimer (Ventana, USA), containing goat derived anti-mouse secondary antibody, at room temperature for 30 min each. Immunoreactivity was visualised using *ultra*View Universal DAB Chromogen (Ventana, USA), containing 3,3′-diaminobenzidine tetrahydrochloride, together with *ultra*View Universal DAB H_2_O_2_ (Ventana, USA), which contains 0.04% hydrogen peroxide (H_2_O_2_) in a phosphate-buffered solution. *ultra*View Universal DAB Copper (Ventana, USA), which contains copper sulfate, was added at room temperature for 3 min to enhance staining intensity. The cells were counterstained with haematoxylin, dehydrated, and mounted with coverslips. Both positive (cisplatin-treated) and negative (serum-free culture medium) controls were included to validate the accuracy and specificity of the staining procedure. The staining assessments were independently verified by Oral Pathology specialists from Faculty of Dentistry, Universiti Kebangsaan Malaysia.

### 4.10. Statistical Analysis

The statistical analysis of data was performed using Statistical Package for the Social Sciences (SPSS) version 29.0 software (IBM Corp, Armonk, New York, NY, USA). All data were presented in mean and standard error of the mean (SEM). Normality of the data distribution was assessed using the Shapiro–Wilk test, with *p* > 0.05 considered indicative of normal distribution. Data were further analysed with one-way between-groups analysis of variance (ANOVA) to test for differences between means of different groups. For post hoc analysis, Tukey’s honestly significant difference (HSD) test was applied when group variances were equal, while Games–Howell analysis was used when variances were unequal. Statistical significance was set at *p* < 0.05.

## 5. Conclusions

CC extract demonstrated significant antiproliferative effects on OSCC cells (IC_50_ = 58 mg/mL) and downregulated *TP53* gene expression with a corresponding reduction in p53 protein, indicating modulation of p53-related pathways. Further studies are required to determine whether these effects involve apoptosis or other cell regulatory mechanisms. CC also showed discordant effects on CD44 (increased mRNA but reduced protein expression), suggesting possible post-transcriptional regulation or selective effects on tumour subpopulations. In contrast, cisplatin exhibited expected cytotoxic and molecular responses, highlighting a different mode of action. Overall, CC shows potential as a complementary therapeutic agent, warranting further studies to elucidate its mechanisms and clinical applicability.

## Figures and Tables

**Table 1:** Nutrient composition of CC extract (per 100 g).

Nutrients	Result(s)
Ash (g)	10.1
Moisture (g)	5.2
Protein (g)	3.7
Total fat (g)	1.2
Carbohydrate (g)	79.8
Energy (kcal)	345
Total flavonoids (mg/g)	35.1
Quercetin (%)	0.77
Polyphenols (mg/kg)	14,900
Vitamin A Retinol (mg)	<0.1
Vitamin C (mg)	1.4
Vitamin E Alpha-Tocopherol (mg)	10

**Table 2:** Primer sequences of *TP53*, *CD44* and housekeeping gene used in this study.

Gene	Accession Number	Product Size	Primer Sequence (5′ to 3′)
*GAPDH*	NM_002046.5	160	F: CAATGACCCCTTCATTGACC R: TTGATTTTGGAGGGATCTCG
*TP53*	NM_000546.6	128	F: GGAGGATGGGGAGTAGGACA R: GCCCCTACCTAGAATGTGGC
*CD44*	NM_001440354.1	166	F: CTACCCCAGCAACCCTACTG R: CTGTCTTCGTCTGGGATGGG

## Data Availability

The original contributions presented in this study are included in the article. Further inquiries can be directed to the corresponding author.

## Abbreviations

The following abbreviations are used in this manuscript:

ANOVA	analysis of variance
CC	*Cosmos caudatus*
cDNA	complementary DNA
CO_2_	carbon dioxide
CSC	cancer stem cell
Ct	threshold cycle
DMEM/F-12	Dulbeccos Modified Eagle Media F-12
DMSO	dimethylsulfoxide
DNA	deoxyribonucleic acid
EDTA	ethylene-diaminetetraacetic acid
ELISA	enzyme-linked immunosorbent assay
FBS	foetal bovine serum
GAPDH	glyceraldehyde 3-phosphate dehydrogenase
H_2_O_2_	hydrogen peroxide
HPV	human papillomavirus
HSD	honestly significant difference
IC_50_	half maximal inhibitory concentration
ICC MDM2	Immunocytochemistry mouse double minute 2 homolog
MTT	thiazolyl blue tetrazolium bromide
NCBI	National Centre for Biotechnology Information
OD	optical density
OSCC	oral squamous cell carcinoma
PCR	polymerase chain reaction
PES	polyethersulfone
PI3K	phosphoinositide 3-kinase
RNA	ribonucleic acid
ROS	reactive oxygen species
RT-qPCR	quantitative reverse transcription polymerase chain reaction
SEM	standard error of the mean
SPSS	Statistical Package for the Social Sciences
TMB	tetramethylbenzidine
TP53	tumour suppressor gene p53
WHO	World Health Organisation

## References

[1] Mahboubi K., Nakoneshny S.C., Sauro K., Roberts S., Hart R., Matthews T.W., Dort J., Chandarana S.P. (2024). The Association of Ethnicity and Oncologic Outcomes for Oral Cavity Squamous Cell Carcinoma (OSCC). Cancers.

[2] Ferlay J., Ervik M., Lam F., Laversanne M., Colombet M., Mery L., Piñeros M., Znaor A., Soerjomataram I., Bray F. Global Cancer Observatory: Cancer Today. https://gco.iarc.who.int/today.

[3] Xu Z., Xu M., Sun Z., Feng Q., Xu S., Peng H. (2025). A nomogram for predicting overall survival in oral squamous cell carcinoma: A SEER database and external validation study. Front. Oncol..

[4] Fatima J., Fatima E., Mehmood F., Ishtiaq I., Khan M.A., Khurshid H.M.S., Kashif M. (2024). Comprehensive Analysis of Oral Squamous Cell Carcinomas: Clinical, Epidemiological, and Histopathological Insights with a Focus on Prognostic Factors and Survival Time. Cureus.

[5] Ahmad P., Nawaz R., Qurban M., Shaikh G.M., Mohamed R.N., Nagarajappa A.K., Asif J.A., Alam M.K. (2021). Risk factors associated with the mortality rate of oral squamous cell carcinoma patients: A 10-year retrospective study. Medicine.

[6] Cabral L.G.d.S., Martins I.M., Paulo E.P.d.A., Pomini K.T., Poyet J.-L., Maria D.A. (2025). Molecular Mechanisms in the Carcinogenesis of Oral Squamous Cell Carcinoma: A Literature Review. Biomolecules.

[7] Miranda-Galvis M., Loveless R., Kowalski L.P., Teng Y. (2021). Impacts of Environmental Factors on Head and Neck Cancer Pathogenesis and Progression. Cells.

[8] Ilyas M., Farhan F., Sadia M., Rehan S.A., Niazi Z., Saeed Z. (2025). Knowledge about Role of HPV as a Risk Factor of Oral Squamous Cell Carcinoma amomg Dentists in Asia: A Systemic Review. J. Khyber Coll. Dent..

[9] Tan Y., Wang Z., Xu M., Li B., Huang Z., Qin S., Nice E.C., Tang J., Huang C. (2023). Oral squamous cell carcinomas: State of the field and emerging directions. Int. J. Oral. Sci..

[10] Lee D.S., Ramirez R.J., Lee J.J., Valenzuela C.V., Zevallos J.P., Mazul A.L., Puram S.V., Doering M.M., Pipkorn P., Jackson R.S. (2021). Survival of Young Versus Old Patients with Oral Cavity Squamous Cell Carcinoma: A Meta-Analysis. Laryngoscope.

[11] Lenoci D., Moresco E., Cavalieri S., Bergamini C., Torchia E., Botta L., Canevari S., Trama A., Licitra L., De Cecco L. (2024). Oral cancer in young adults: Incidence, risk factors, prognosis, and molecular biomarkers. Front. Oncol..

[12] Cheng H.-Y., Wu Y.-X., Yu Z.-L. (2025). Unique clinical features and prognostic risk factors of oral squamous cell carcinoma in patients under 30 years old. Clin. Oral. Investig..

[13] Hong X., Quimby A.E., Mavedatnia D., Pickett A.T., Corsten M., Zhang T., Tohme A., Johnson-Obaseki S., Khalil C., Khoury M. (2025). Oral Cavity Squamous Cell Carcinoma in Young Patients: A Multi-Institutional Study of the Canadian Head & Neck Collaborative Research Initiative. J. Otolaryngol.-Head Neck Surg..

[14] Mneimneh W.S., Xu B., Ghossein C., Alzumaili B., Sethi S., Ganly I., Khimraj A., Dogan S., Katabi N. (2021). Clinicopathologic Characteristics of Young Patients with Oral Squamous Cell Carcinoma. Head Neck Pathol..

[15] Sasaki K., Takahashi S., Ouchi K., Otsuki Y., Wakayama S., Ishioka C. (2023). Different impacts of TP53 mutations on cell cycle-related gene expression among cancer types. Sci. Rep..

[16] Ali S.A., Khan H.A., Irfan O., Samad A., Mirza Y., Awan M.S. (2017). Correlation of TP53 Overexpression and Clinical Parameters with Five-Year Survival in Oral Squamous Cell Carcinoma Patients. Cureus.

[17] Gawande M., Chaudhary M., Sharma P., Hande A., Patil S., Sonone A. (2020). Expression of p53 at invasive front of oral squamous cell carcinoma and negative histopathological surgical margins to establish correlation at 3-year survival. J. Oral. Maxillofac. Pathol..

[18] Puteri A., Meizarini A. (2020). p53: The guardian of genome against OSCC progression. Eur. J. Mol. Clin. Med..

[19] Chu X., Tian W., Ning J., Xiao G., Zhou Y., Wang Z., Zhai Z., Tanzhu G., Yang J., Zhou R. (2024). Cancer stem cells: Advances in knowledge and implications for cancer therapy. Signal Transduct. Target. Ther..

[20] Shahoumi L.A. (2021). Oral Cancer Stem Cells: Therapeutic Implications and Challenges. Front. Oral. Health.

[21] Nanda Kumar H., Vasanthi V., Gunasekaran N., Divya B., Annasamy R.K., Krishnan R. (2024). Correlation of prognosis of oral squamous cell carcinoma with CD44 expression: A retrospective immunohistochemical analysis. Cancer Res. Stat. Treat..

[22] Singh B., Aggarwal S., Das P., Srivastava S.K., Sharma S.C., Das S.N. (2023). Over Expression of Cancer Stem Cell Marker CD44 and Its Clinical Significance in Patients with Oral Squamous Cell Carcinoma. Indian J. Otolaryngol. Head Neck Surg..

[23] Mirhashemi M., Sadeghi M., Ghazi N., Saghravanian N., Dehghani M., Aminian A. (2023). Prognostic value of CD44 expression in oral squamous cell carcinoma: A meta-analysis. Ann. Diagn. Pathol..

[24] Miao S., Wang C., Zheng A. (2025). CD44 overexpression as a mediator of drug resistance in oral cancer: A meta-analysis unveiling molecular underpinnings and therapeutic implications. Discov. Oncol..

[25] Petrović A., Mojsilović S., Bugarski D., Jauković A., Pokimica B., Ilić M.P. (2025). Expression of Putative Cancer Stem Cell Markers in Oral Squamous Cell Carcinoma: Correlation with Clinicopathological Features. Int. J. Mol. Sci..

[26] Melo-Alvim C., Neves M.E., Santos J.L., Abrunhosa-Branquinho A.N., Barroso T., Costa L., Ribeiro L. (2022). Radiotherapy, Chemotherapy and Immunotherapy-Current Practice and Future Perspectives for Recurrent/Metastatic Oral Cavity Squamous Cell Carcinoma. Diagnostics.

[27] Shetty R.K., Pradhan S., Kannan R., Doctor A., Surnare K., Jondhale M., Patil D., Shetty N. (2020). Clinical Profile and Quality of Life Assessment of Oral Cancer Patients Following Nasolabial Flap Reconstruction Surgery. Indian J. Otolaryngol. Head Neck Surg..

[28] Feller G., Ramiah D., Mahomed F., Feller L., Khammissa R.A.G. (2025). Radiotherapy and Its Consequences in Relation to Oral Squamous Cell Carcinoma—A Narrative Review. Radiation.

[29] Kasahara Y., Endo K., Ueno T., Ueno H., Moriyama-Kita M., Odani A., Yoshizaki T. (2019). Bone invasion-targeted chemotherapy with a novel anionic platinum complex (3Pt) for oral squamous cell carcinoma. Cancer Sci..

[30] Zhou Y., Wang L., Liu M., Jiang H., Wu Y. (2025). Oral squamous cell carcinoma: Insights into cellular heterogeneity, drug resistance, and evolutionary trajectories. Cell Biol. Toxicol..

[31] Kujan O., van Schaijik B., Farah C.S. (2020). Immune Checkpoint Inhibitors in Oral Cavity Squamous Cell Carcinoma and Oral Potentially Malignant Disorders: A Systematic Review. Cancers.

[32] Situmorang P.C., Ilyas S., Nugraha S.E., Syahputra R.A., Nik Abd Rahman N.M.A. (2024). Prospects of compounds of herbal plants as anticancer agents: A comprehensive review from molecular pathways. Front. Pharmacol..

[33] Reinhardt K., Hood Salleh R.S.H., Jaenicke C., Gruenwald J., Abidin Z.Z. (2016). Health Beauty from the Rainforest Malaysian Traditions of Ramuan.

[34] Alhakam A.A., Shahidan W.N.S. (2021). *Cosmos caudatus*: A Possible Drug Candidate for Oral Squamous Cell Carcinoma. Curr. Bioact. Compd..

[35] Moshawih S., Cheema M., Ahmad Z., Zakaria Z.A., Hakim M. (2017). A Comprehensive Review on *Cosmos caudatus* (Ulam Raja): Pharmacology, Ethnopharmacology, and Phytochemistry. Int. Res. J. Educ. Sci..

[36] Sandra F., Rizal M., Dhaniar A., Scania A., Lee K. (2024). *Cosmos caudatus* Leaf Extract Triggers Apoptosis of HSC-3 Cancer Cells by Decreasing Bcl-2 and Increasing Bax. Indones. Biomed. J..

[37] Uzbek U., Shahidan W. (2019). Tasty Herb that Heals: A Review of *Cosmos caudatus* (Ulam Raja) and its Potential Uses in Dentistry. World J. Dent..

[38] Yusoff N.A.H., Rukayadi Y., Abas F., Khatib A., Hassan M. (2021). Antimicrobial stability of *Cosmos caudatus* extract at varies pH and temperature, and compounds identification for application as food sanitiser. Food Res..

[39] Molina-Montes E., Salamanca-Fernández E., Garcia-Villanova B., Sánchez M.J. (2020). The Impact of Plant-Based Dietary Patterns on Cancer-Related Outcomes: A Rapid Review and Meta-Analysis. Nutrients.

[40] Menegassi B., Vinciguerra M. (2025). Ultraprocessed Food and Risk of Cancer: Mechanistic Pathways and Public Health Implications. Cancers.

[41] Jun S., Park H., Kim U.J., Choi E.J., Lee H.A., Park B., Lee S.Y., Jee S.H., Park H. (2023). Cancer risk based on alcohol consumption levels: A comprehensive systematic review and meta-analysis. Epidemiol. Health.

[42] Nurhayati B., Rahayu I.G., Rinaldi S.F., Zaini W.S., Afifah E., Arumwardana S., Kusuma H.S.W., Rizal R., Widowati W. (2018). The Antioxidant and Cytotoxic Effects of *Cosmos caudatus* Ethanolic Extract on Cervical Cancer. Indones. Biomed. J..

[43] Pebriana R., Wardhani B., Wardhani K., Widayanti E., Latifah N., Wijayanti S., Wijayanti T., Riyanto S., Meiyanto E. (2008). Apoptotic Effect of Kenikir Leaves (*Cosmos caudatus* Kunth.) Methanolic Extract on Breast Cancer Cell Line. Pharmaco.

[44] Ramadhan F., Mukarramah L., Risma F., Yulian R., Hilyatun N., Asyiah I. (2018). Flavonoids from endophytic bacteria of *Cosmos caudatus* Kunth. Leaf as anticancer and antimicrobial. Asian J. Pharm. Clin. Res..

[45] Huang C.F., Liu S.H., Ho T.J., Lee K.I., Fang K.M., Lo W.C., Liu J.M., Wu C.C., Su C.C. (2022). Quercetin induces tongue squamous cell carcinoma cell apoptosis via the JNK activation-regulated ERK/GSK-3α/β-mediated mitochondria-dependent apoptotic signaling pathway. Oncol. Lett..

[46] Fita F.E., Listianingsih D., Hapsari Y.A., Pradana R.G., Safitri E.I., Arifin I. (2015). Efek Sitotoksik Kombinasi Ekstrak Metanol Daun Kenikir (*Cosmos caudatus*, Kunth) dan Doksorubisin Terhadap Sel Kanser Payudara T47D Secara In-Vitro dan In-Silico. Prosiding Seminar Nasional “Peluang Herbal Sebagai Alternative Medicine”. J. Ilmu Farm. Dan Farm. Klin..

[47] Cheriyan B., Pandi K., Murali D., Boobalan N., Harikrishnan J., Kannan D., Jebaraj L., Munuswamy S., Manaksha S. (2025). Polyphenols Target Apoptosis and Cell Cycle Regulation in Various Cancer Models: Emphasis on Flavonoid Subclasses. Biomed. Pharmacol. J..

[48] Hyodo T., Kuribayashi N., Fukumoto C., Komiyama Y., Shiraishi R., Kamimura R., Sawatani Y., Yaguchi E., Hasegawa T., Izumi S. (2022). The mutational spectrum in whole exon of p53 in oral squamous cell carcinoma and its clinical implications. Sci. Rep..

[49] Umar A.H., Biringallo C.S.N., Tuyuwale P.I., Kila A., Febyola K.D., Syahruni R., Hendrarti W., Rafi M., Ratnadewi D. (2025). Unveiling the Pharmacological Mechanism of *Cosmos caudatus* Compounds as Lung Cancer Drug Candidates: Pharmacology Networking, Molecular Docking, and Experimental Validation. J. Pharm. Innov..

[50] S Reihani S.F., Tan T.-C., Alkarkhi A., Easa A. (2016). Total Phenolic Content and Antioxidant Activity of Ulam Raja (*Cosmos caudatus*) and Quantification of its Selected Marker Compounds: Effect of Extraction. Int. J. Food Prop..

[51] Jakobek L. (2015). Interactions of polyphenols with carbohydrates, lipids and proteins. Food Chem..

[52] Wróblewski M., Wróblewska W., Sobiesiak M. (2024). The Role of Selected Elements in Oxidative Stress Protection: Key to Healthy Fertility and Reproduction. Int. J. Mol. Sci..

[53] Hamdi N.A.M., Haris M.S., Ismail A.F.H. (2021). The positive impact of Vitamin C (Ascorbic Acid) utilisation in cancer treatment: A scoping review of published articles from the perspective of the in vitro studies. Malays. J. Med. Health Sci..

[54] You A.J., Park J., Shin J.-M., Kim T.H. (2025). Oxidative Stress and Dietary Antioxidants in Head and Neck Cancer. Antioxidants.

[55] Zhou J., Chen C., Chen X., Fei Y., Jiang L., Wang G. (2020). Vitamin C Promotes Apoptosis and Cell Cycle Arrest in Oral Squamous Cell Carcinoma. Front. Oncol..

[56] Mariana Florica B., Daniela D., Gheorghe S., Daniela Margareta V., Mihaela Dana P. (2023). Influence of Vitamins and Antioxidants in Oral Carcinogenesis—A Review. Pharmacophore.

[57] Andrés C.M., Pérez de la Lastra J.M., Juan C.A., Plou F.J., Pérez-Lebeña E. (2023). Polyphenols as Antioxidant/Pro-Oxidant Compounds and Donors of Reducing Species: Relationship with Human Antioxidant Metabolism. Processes.

[58] Zhou Y., Jiang Z., Lu H., Xu Z., Tong R., Shi J., Jia G. (2019). Recent Advances of Natural Polyphenols Activators for Keap1-Nrf2 Signaling Pathway. Chem. Biodivers..

[59] Granado-Serrano A.B., Martín M.A., Bravo L., Goya L., Ramos S. (2012). Quercetin modulates Nrf2 and glutathione-related defenses in HepG2 cells: Involvement of p38. Chem.-Biol. Interact..

[60] Qi W., Qi W., Xiong D., Long M. (2022). Quercetin: Its Antioxidant Mechanism, Antibacterial Properties and Potential Application in Prevention and Control of Toxipathy. Molecules.

[61] Xu D., Hu M.J., Wang Y.Q., Cui Y.L. (2019). Antioxidant Activities of Quercetin and Its Complexes for Medicinal Application. Molecules.

[62] Cheng S.H., Barakatun-Nisak M.Y., Anthony J., Ismail A. (2015). Potential medicinal benefits of *Cosmos caudatus* (Ulam Raja): A scoping review. J. Res. Med. Sci..

[63] Anttila J.V., Shubin M., Cairns J., Borse F., Guo Q., Mononen T., Vázquez-García I., Pulkkinen O., Mustonen V. (2019). Contrasting the impact of cytotoxic and cytostatic drug therapies on tumour progression. PLoS Comput. Biol..

[64] Magnano S., Hannon Barroeta P., Duffy R., O’Sullivan J., Zisterer D.M. (2021). Cisplatin induces autophagy-associated apoptosis in human oral squamous cell carcinoma (OSCC) mediated in part through reactive oxygen species. Toxicol. Appl. Pharmacol..

[65] Silva L.C., Leite A.A., Borgato G.B., Wagner V.P., Martins M.D., Loureiro F.J.A., Lopes M.A., Santos-Silva A.R., Sperandio M., de Castro Junior G. (2023). Oral squamous cell carcinoma cancer stem cells have different drug sensitive to pharmacological NFκB and histone deacetylation inhibition. Am. J. Cancer Res..

[66] Khoo X.H., Paterson I.C., Goh B.H., Lee W.L. (2019). Cisplatin-Resistance in Oral Squamous Cell Carcinoma: Regulation by Tumor Cell-Derived Extracellular Vesicles. Cancers.

[67] Arokia Femina T., Barghavi V., Archana K., Swethaa N.G., Maddaly R. (2023). Non-uniformity in in vitro drug-induced cytotoxicity as evidenced by differences in IC50 values—Implications and way forward. J. Pharmacol. Toxicol. Methods.

[68] Wang H., Guo M., Wei H., Chen Y. (2023). Targeting p53 pathways: Mechanisms, structures and advances in therapy. Signal Transduct. Target. Ther..

[69] Tornesello M.L. (2025). TP53 mutations in cancer: Molecular features and therapeutic opportunities (Review). Int. J. Mol. Med..

[70] Shi T., Polderman P.E., Burgering B.M.T., Dansen T.B. (2020). DNA damage and oxidizing conditions activate p53 through differential upstream signaling pathways. bioRxiv.

[71] Liu S., Yang W., Li Y., Sun C. (2023). Fetal bovine serum, an important factor affecting the reproducibility of cell experiments. Sci. Rep..

[72] Mun S.E., Sim B.W., Yoon S.B., Jeong P.S., Yang H.J., Choi S.A., Park Y.H., Kim Y.H., Kang P., Jeong K.J. (2017). Dual effect of fetal bovine serum on early development depends on stage-specific reactive oxygen species demands in pigs. PLoS ONE.

[73] Zhang Y., Xu Y.-Y., Sun W., Zhang M.-H., Zheng Y.-F., Shen H.-M., Yang J., Zhu X.-Q. (2016). FBS or BSA Inhibits EGCG Induced Cell Death through Covalent Binding and the Reduction of Intracellular ROS Production. BioMed. Res. Int..

[74] Liu B., Chen Y., St Clair D.K. (2008). ROS and p53: A versatile partnership. Free Radic. Biol. Med..

[75] Yaghobi Z., Movassaghpour A., Talebi M., Abdoli Shadbad M., Hajiasgharzadeh K., Pourvahdani S., Baradaran B. (2021). The role of CD44 in cancer chemoresistance: A concise review. Eur. J. Pharmacol..

[76] Koh Y.C., Ho C.T., Pan M.H. (2020). Recent advances in cancer chemoprevention with phytochemicals. J. Food Drug Anal..

[77] Chen C., Zhao S., Karnad A., Freeman J.W. (2018). The biology and role of CD44 in cancer progression: Therapeutic implications. J. Hematol. Oncol..

[78] Ohkoshi E., Umemura N. (2017). Induced overexpression of CD44 associated with resistance to apoptosis on DNA damage response in human head and neck squamous cell carcinoma cells. Int. J. Oncol..

[79] Wu Z., Lu J., Loo A., Ho N., Nguyen D., Cheng P.Y., Mohammed A.I., Cirillo N. (2024). Role of CD44 in Chemotherapy Treatment Outcome: A Scoping Review of Clinical Studies. Int. J. Mol. Sci..

[80] Qiao X., Zhu L., Song R., Shang C., Guo Y. (2023). CD44 occurring alternative splicing promotes cisplatin resistance and evokes tumor immune response in oral squamous cell carcinoma cells. Transl. Oncol..

[81] Li M., Sun D., Song N., Chen X., Zhang X., Zheng W., Yu Y., Han C. (2023). Mutant p53 in head and neck squamous cell carcinoma: Molecular mechanism of gain-of-function and targeting therapy (Review). Oncol. Rep..

[82] Jo D.W., Kim Y.K., Yun P.Y. (2016). The influence of p53 mutation status on the anti-cancer effect of cisplatin in oral squamous cell carcinoma cell lines. J. Korean Assoc. Oral. Maxillofac. Surg..

[83] Buja L.M. (2021). The cell theory and cellular pathology: Discovery, refinements and applications fundamental to advances in biology and medicine. Exp. Mol. Pathol..

[84] Adnan Y., Ali S.M.A., Farooqui H.A., Kayani H.A., Idrees R., Awan M.S. (2022). High CD44 Immunoexpression Correlates with Poor Overall Survival: Assessing the Role of Cancer Stem Cell Markers in Oral Squamous Cell Carcinoma Patients from the High-Risk Population of Pakistan. Int. J. Surg. Oncol..

[85] Khan A.S., Ahmad S., Iqbal F., Saboor A., Nisar M., Naushin T., Khalid Sheikh A., Haq M., Ahmad T., Rehman B. (2022). A Immunohistochemical Expression of p53 in Oral Squamous Cell Carcinoma, Oral Epithelial Precursor Lesions, and Normal Oral Mucosa. J. Med. Sci..

[86] Ahda M., Jaswir I., Khatib A., Ahmed Q.U., Syed Mohamad S.N.A. (2023). A review on *Cosmos caudatus* as A potential medicinal plant based on pharmacognosy, phytochemistry, and pharmacological activities. Int. J. Food Prop..

[87] Hashim G.M., Shahgolzari M., Hefferon K., Yavari A., Venkataraman S. (2025). Plant-Derived Anti-Cancer Therapeutics and Biopharmaceuticals. Bioengineering.

[88] Martin T.M., Kishore Kumar M.S. (2025). Chemoprotective Horizons: Bioactive Molecules as Therapeutic Shields Against Cytotoxicity. Asian Pac. J. Cancer Biol..

[89] Lozano G., Prives C., Sabapathy K. (2025). Mutant p53 Gain of Function: Why Many See It, Why Some Do Not. Cancer Discov..

[90] Chen X., Zhang T., Su W., Dou Z., Zhao D., Jin X., Lei H., Wang J., Xie X., Cheng B. (2022). Mutant p53 in cancer: From molecular mechanism to therapeutic modulation. Cell Death Dis..

[91] Wang J., Liu W., Zhang L., Zhang J. (2023). Targeting mutant p53 stabilization for cancer therapy. Front. Pharmacol..

[92] Lekhak N., Bhattarai H.K. (2024). Phytochemicals in Cancer Chemoprevention: Preclinical and Clinical Studies. Cancer Control.

[93] Rao M.J., Duan M., Zhou C., Jiao J., Cheng P., Yang L., Wei W., Shen Q., Ji P., Yang Y. (2025). Antioxidant Defense System in Plants: Reactive Oxygen Species Production, Signaling, and Scavenging During Abiotic Stress-Induced Oxidative Damage. Horticulturae.

[94] Ghasemi M., Turnbull T., Sebastian S., Kempson I. (2021). The MTT Assay: Utility, Limitations, Pitfalls, and Interpretation in Bulk and Single-Cell Analysis. Int. J. Mol. Sci..

[95] Robles-Espinoza C.D., Mohammadi P., Bonilla X., Gutierrez-Arcelus M. (2021). Allele-specific expression: Applications in cancer and technical considerations. Curr. Opin. Genet. Dev..

[96] Barber A.E., Meek D.W. (2021). Detection of Post-translationally Modified p53 by Western Blotting. Methods Mol. Biol..

[97] Yang C.Y., Meng C.L. (1994). Regulation of PG synthase by EGF and PDGF in human oral, breast, stomach, and fibrosarcoma cancer cell lines. J. Dent. Res..

[98] Camacho-Alonso F., Gómez-Albentosa T., Oñate-Sánchez R.E., Tudela-Mulero M.R., Sánchez-Siles M., Gómez-García F.J., Guerrero-Sánchez Y. (2020). In Vitro Study of Synergic Effect of Cisplatin and Low Molecular Weight Heparin on Oral Squamous Cell Carcinoma. Front. Oncol..

[99] Louis K.S., Siegel A.C. (2011). Cell viability analysis using trypan blue: Manual and automated methods. Methods Mol. Biol..

[100] (2009). Biological Evaluation of Medical Devices—Part 5: Tests for In Vitro Cytotoxicity.

[101] Zhu X., Park S., Lee W.K., Cheng S.Y. (2019). Potentiated anti-tumor effects of BETi by MEKi in anaplastic thyroid cancer. Endocr. Relat. Cancer.

[102] Wu Z., Wang C., Han J., Chen X., Wu J., Cheng B., Wang J. (2025). Exploring Renin-angiotensin System Genes as Novel Prognostic Biomarkers for Oral Squamous Cell Carcinoma. Int. J. Med. Sci..

